# Geographic Access to Primary Healthcare Services among Latinos/as/x in Western Alabama

**DOI:** 10.14423/SMJ.0000000000001476

**Published:** 2022-12

**Authors:** Mercedes M. Morales-Alemán, Qinglin Hu, Gwendolyn Ferreti, Lea G. Yerby

**Affiliations:** Department of Community Medicine and Population Health and the Institute for Rural Health Research, University of Alabama, Tuscaloosa; and the Department of Peace and Social Justice Studies, Berea College, Berea, Kentucky.

**Keywords:** geographic healthcare access, GIS, Latinos, US South

## Abstract

**Objectives:**

Alabama’s Latino/a/x population grew 278% from 2000 to 2018. Tuscaloosa County, located in the largely rural region of western Alabama, also experienced a significant influx of Latino/as/x during this time frame. Geographic healthcare access (GHA) to primary care and hospitals is crucial for immigrant Latino/as/x to care for their health, but few studies have characterized it. The goals of this article were to describe the availability (defined as number of provider locations) and accessibility (defined as travel impedance between potential patients and provider locations) of primary healthcare services and to discuss potential strategies to address these healthcare access challenges.

**Methods:**

We drew data from the US Census Bureau, American Community Survey 5-year estimates, Blue Cross Blue Shield national doctor and hospital finder database, and the Alabama Department of Public Health and Tuscaloosa Transit Authority. We used geographic data, geographic information systems, and spatial analyses to characterize the availability and accessibility of primary care services and hospitals for Latinos/as/x in Tuscaloosa County using ESRI, ArcGIS 10.6.1. We showed the distribution of Latinos/as/x by census tract with choropleth mapping and mapped primary healthcare providers alongside public transit routes and hospital driving times to support our findings.

**Results:**

This work demonstrated that Latinos/as/x in Tuscaloosa County were concentrated in more rural areas surrounding the county’s city center, presenting significant barriers to geographic healthcare access. These areas had fewer primary care providers and limited public transit. Many Latinos/as/x in this county had to travel ≥45 minutes to a hospital.

**Conclusions:**

Outreach and technology-based approaches, including home visit programs, mobile health units, and telemedicine, may be particularly important in bridging the geographic healthcare access gaps for this and other largely rural populations the southeastern United States. Some of this potential was unlocked during the coronavirus disease 2019 crisis. These gains should be leveraged toward sustainable healthcare access initiatives for rural Latino/a/x populations.

The Latino/a/x population is the largest ethnic minority group in the United States.^1^ Empirical evidence shows that Latinos/as/x in the United States are disproportionately affected by adverse social determinants of health (ie, poor conditions of daily life shaped by structural and social position factors).^2^ In addition, epidemiological data demonstrates that Latinos/as/x are disproportionately affected by chronic health conditions that require primary care management, including diabetes, asthma, and heart disease.^3^

## Defining Terms

Hispanic and Latina/o are ethno-racial categories used to describe individuals of Latin American descent and often are used interchangeably. The former emphasizes language and the latter region. The term Latinx is a gender-neutral term meant to center the broad range of gender identities present among individuals of Latin American descent.^4^ We use Latina/o/x in this article; however, some of our data sources use the term Hispanic.^1^ In those instances, we have preserved the terms used by the original data sources for accuracy and consistency.

## Latinos/as/x in Alabama

Between 2000 and 2018, Alabama was the state with the fastest growing Latino/a/x population in the United States. The Latino/a/x population grew by 278% during that time.^5^ As of 2018, Latinos/as/x made up an estimated 4.3% of Alabama’s population.^6^ Tuscaloosa County, located in the largely rural region of western Alabama, also experienced a significant influx of Latino/as/x during this time.^7^ In 2018, Latinos/as/x represented 3.9% of the total population in Tuscaloosa County.^7^ Research conducted by our group shows that Latinos/as/x in western Alabama face significant structural challenges to healthcare access, including transportation and language access.^8,9^ Furthermore, some Latinos/as/x populations in Alabama report worse health than their counterparts in other parts of the country.^10^

Geographic healthcare access (GHA) (defined as the degree to which healthcare services are spatially available to geographically defined groups) to primary care and hospital-based care is crucial for communities to care for their health^11–13^ and is a predictor of healthcare utilization.^14–16^ A significant proportion of Latinos/as/x who migrated and settled in southern US states at the beginning of the 21st century live in rural areas where GHA to primary healthcare services and hospitals often is inadequate.^17,18^ Travel impedances such as long driving times and few public transportation options present barriers to healthcare access for these populations^19,20^ Few studies have sought to describe GHA to primary health care in emerging Latino/a/x regions such as the US South, however. The goals of this study are to describe the availability (defined as number of provider locations) and accessibility (defined as travel impedance between potential patients and provider locations)^21^ of primary healthcare services in Tuscaloosa County, identify geographic healthcare gaps for Latino/a/x populations in this region, and discuss potential strategies to address these healthcare access challenges.

## Methods

### Study Setting

Tuscaloosa County is the sixth most populous county in Alabama.^6^ It had a population of 206,213 in 2018.^6,7^ Approximately 65% of the population in Tuscaloosa County is White, 32% is African American, and 3.9% is Hispanic.^6^ Geographically, Tuscaloosa County is 1321.7 mi^2^, the third largest county in Alabama.^6^ Almost 75% of the Tuscaloosa County population live in the centrally located urban area (the city of Tuscaloosa) and 25.5% of the population lives in the surrounding rural parts of the county.^6^ Tuscaloosa County is largely rural based on a population density of 153 people per square mile.^22^ The only city in Tuscaloosa County with more than 500 people per square mile is the city of Tuscaloosa (total population 98,881 in 2018).^6^

With regard to the healthcare landscape, Tuscaloosa County has two hospitals. In addition, Tuscaloosa County has a primary care population-to-primary care physician ratio of 1378:1, compared with 1530:1 at the state level.^23^ It is considered by the Health Resources and Services Administration to be a Health Professional Shortage Area in primary care, dental care, and mental health because of its designation as a low-income area.^24^

### Data Sources

We drew the data for the spatial analyses described in this study from the US Census Bureau 2014–2018 American Community Survey (ACS) 5-year estimates^7^ (socioeconomic variables), the 2018 Blue Cross Blue Shield (BCBS) national doctor and hospital finder database (provider specialty and location data),^25^ the Alabama Department of Public Health (Medicaid and hospital data), and the Tuscaloosa Transit Authority (geocoded transit route data).^26^ The ACS is a nationwide survey conducted every 5 years that provides publicly demographic information for use by residents, researchers, and government entities.^7^ The BCBS national doctor and hospital finder database provides the location and specialty information for healthcare providers in the United States who accept BCBS. (We chose the BCBS national doctor and hospital finder as the data source for providers who accepted private insurance because, as of 2018, BCBS of Alabama controlled 94% of the insurance market in Alabama.^27,28^) We accessed the data on Medicaid providers from the Patient 1st Program, the managed care program for primary services operated by the Alabama Medicaid Agency at the time of these analyses. Finally, we gathered the data on hospitals from the Alabama Department of Public Health.

### Geographic Information Systems (GIS) Analyses

**Figure 1.**
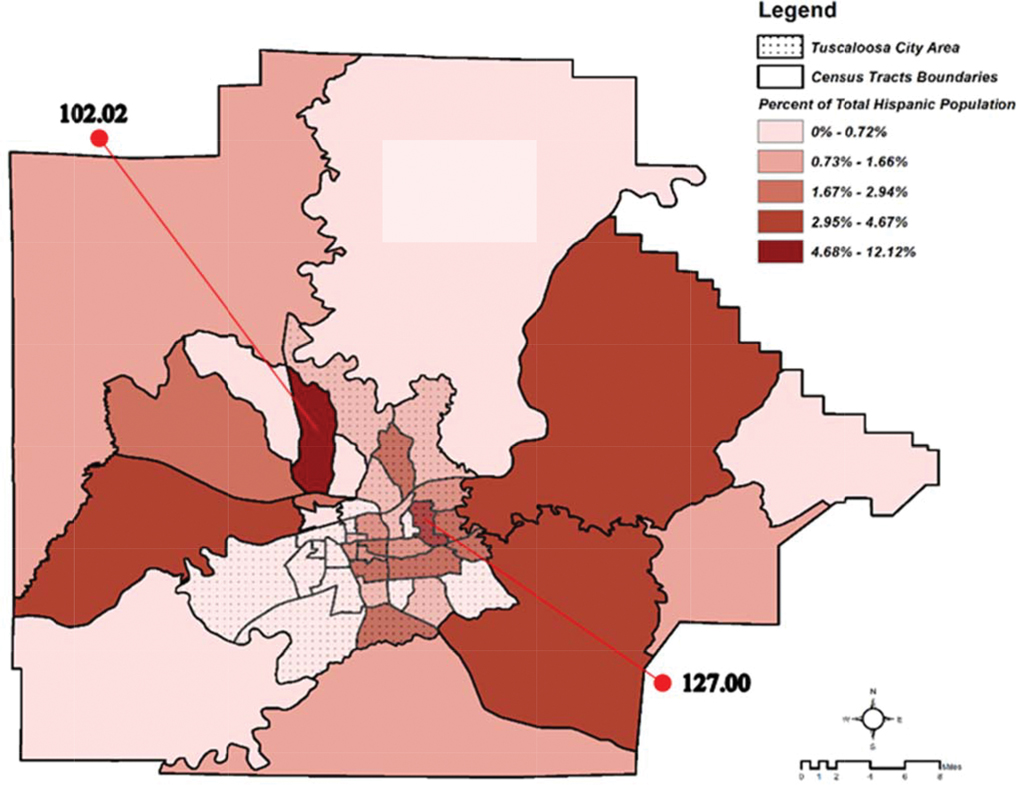
Percent of the Total population Composed of Hispanics/Latino/a/xs by Census Tract

**Figure 3.**
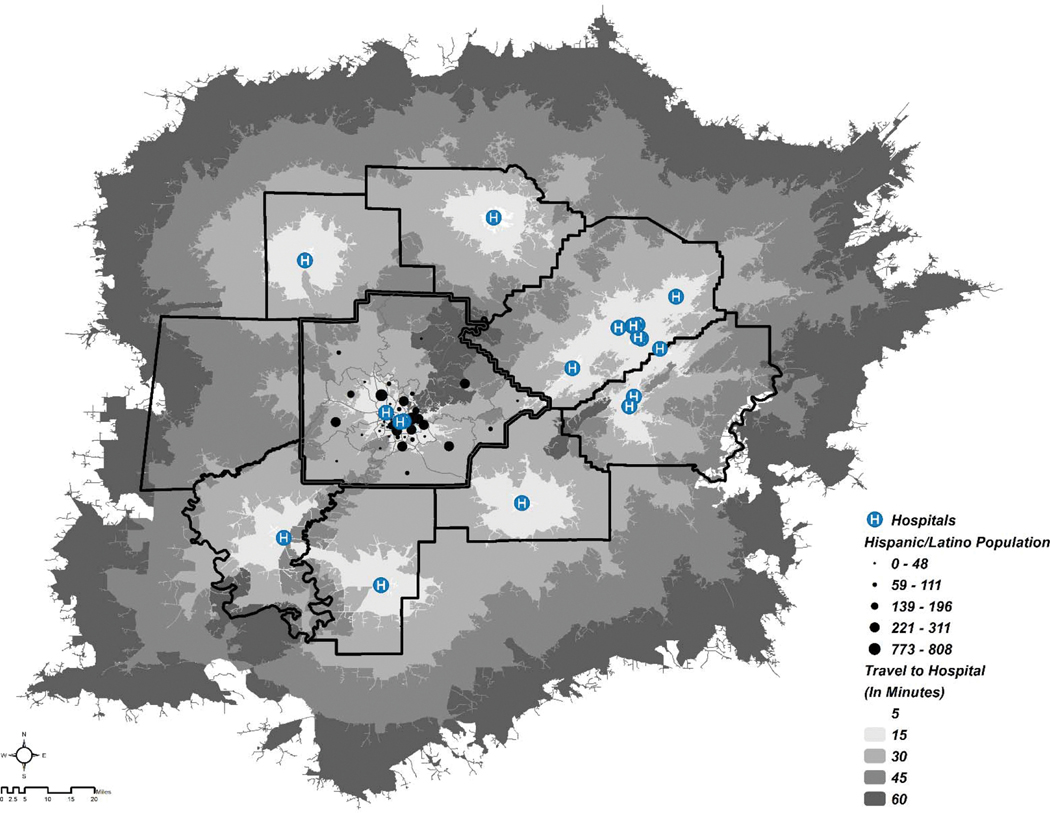

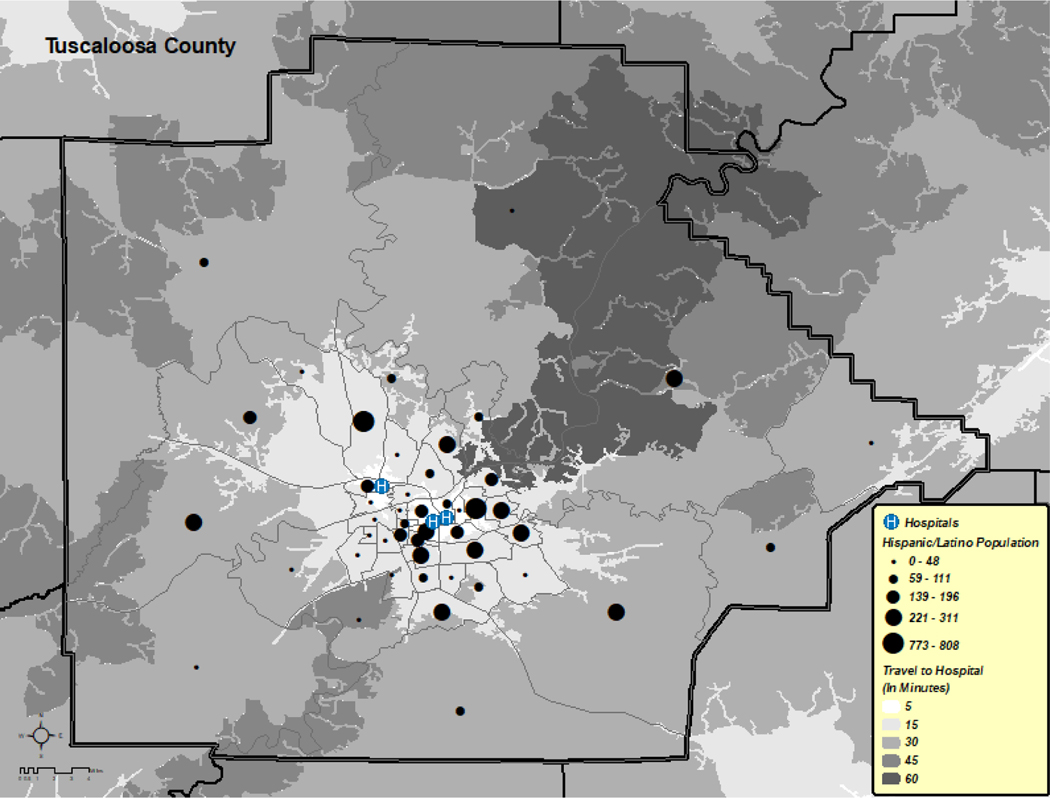
Driving Times to Hospitals

GIS and spatial analyses were used to characterize the availability and accessibility of primary care services for Latinos/as/x in Tuscaloosa County. The statistical unit of analysis for this study was the census tract, defined as a small, relatively permanent statistical subdivision of a county or equivalent entity established by the US Census Bureau.^29^ We used Choropleth mapping with natural breaks (Jenks) classification^30^ to visualize the geospatial distribution of the Latino/a/x population in each tract, an appropriate methodology when mapping unevenly distributed data such as population density.^31^ Tracts were shaded proportionally to their assigned values on the map to allow the visualization of geospatial variation. We mapped the following:

1. The density of the Latino/a/x population in Tuscaloosa County by US census tract (Appendix I, Figure 1, [COMP: INSERT SDC1 URL HERE]).
2. Primary care service providers by subspecialty (specifically, family practitioner, internal medicine, and general pediatrician; Appendix I, Figure 2a, 2c, and 2e). The types of health insurance plans that they accepted (Medicaid or Blue Cross Blue Shield of Alabama), and city of Tuscaloosa public transit routes (Appendix I, Figure 2b, 2d, and 2f).
3. Driving times to hospitals for Latinos/as/x living in Tuscaloosa County (Appendix I, Figure 3).

We manually collected and geocoded the primary care provider and transit route data into GIS^26^ using ESRI software, ArcGIS 10.6.1^32^ to map the distribution of primary healthcare providers alongside public transit accessibility. We used two different symbols to illustrate the locations of provider facilities by the types of insurance plans that they accepted (stars indicate Medicaid providers and crosses indicate BCBS of Alabama providers) (Appendix I, Figure 2b, 2d, and 2f). Public transit maps show the public transit bus routes, which only operate within the city limits. Each dotted line represents a different public transit route operated in Tuscaloosa County. According to the Tuscaloosa Transit Authority route schedule, the average time interval between two buses is approximately 1 hour.

### Driving Time to Hospitals Map (Appendix I, Figure 3)

To illustrate the accessibility to hospitals for Latinos/as/x in Tuscaloosa County, we derived driving time catchments based on the Tuscaloosa County road network system and the locations of hospitals (Appendix I, Figure 3).^33^ For the purposes of this article, we used the Alabama Department of Public Health definition of a hospital: “A health institution planned, organized, and maintained for offering to the public, generally, facilities and beds for use in the diagnosis and/or treatment of illness, disease, injury, deformity, abnormality or pregnancy, when the institution offers such care or service for not less than 24 consecutive hours in any week to two or more individuals not related by blood or marriage to the owner and/or chief executive officer/administrator.”^34^ We collected and georeferenced the hospital location data in each map overlaid with the Latinos/as/x population at the census tract level for visualization and analysis (Appendix I, Figure 3). We used a network analysis module to create a road network and calculate proximity within a maximum catchment of 60-minute driving time from the Latinos/as/x residential areas to the geocoded hospitals.^33^ This approach has been used widely in previous works to calculate travel time and to measure spatial accessibility.^11,35–37^ We used travel time as the measure of impedance (which attempts to capture the passengers’ behavior and their subjective perceptions of impedance when traveling in the transit networks) by combining travel impact variables and road travel segments.^38^ The travel impact variables usually include speed limit, historical travel time, peak/off-peak hours, weather conditions, and daytime/nighttime. The road travel segments represent the road type, highway, or local road. This allowed for an accurate prediction of travel time between any two points that were connected by the road networks. Driving time catchments were produced for hospitals at time intervals of 5 to 15 minutes. We set a 1-hour travel time as our study time cap because, based on previous studies, it reflects a reasonable daily commute time for most patients.^33^

## Results

Tables 1 and 2 in Appendix II ([COMP: INSERT SDC2 URL HERE]) include selected demographic characteristics of Latinos/as/x in Tuscaloosa County and in Alabama derived from the 2014–2018 ACS 5-year estimates.^7^ Overall, 32% of Latinos/as/x in Alabama lived below the poverty level ($25,100) and 25% of Latinos/as/x in Tuscaloosa County lived below the poverty level.^6^ The annual median income for Latinos/as/x in Alabama was $37,412 and Tuscaloosa County was $23,212 for Latinos/as/x, 38% lower than the state level. With regard to education, 6% of Latinos/as/x in Alabama and 5% of Latinos/as/x in Tuscaloosa County had a Bachelor’s degree or higher.^7^ With regard to language, 29% of Latinos/as/x in Alabama and 27% in Tuscaloosa County reported that they spoke English “not well” or “not at all.” An estimated 29% of Latinos/as/x in Alabama and 28% of Latinos/as/x in Tuscaloosa County were uninsured in 2018.^39^

Table 3 in Appendix II indicates the numbers of primary care service providers by subspecialty (specifically, Family Practitioner, Internal Medicine, and General Pediatrician) and the types of health insurance plans that they accepted (Medicaid or BCBS of Alabama) by 2018 (Blue Cross Blue Shield Association, 2018, Medicaid, and hospital data). All three subspecialty providers in Tuscaloosa County were surveyed and analyzed in our study. The fact is that not all of them were accepting Medicaid patients, which implies that Latino/as/x or other population groups who were enrolled in the Medicaid program may not receive treatment from their geographically closest providers.

The map in Figure 1 of Appendix I ([COMP: INSERT SDC1 URL HERE]) depicts the spatial distribution of the Latino/a/x population in Tuscaloosa County by census tract. Figure 1 of Appendix I illustrates the percentage of the total population that was composed of Latinos/as/x by census tract. Two tracts (127.00 and 102.02) showed a significant proportion of Latinos/as/x (4.68%–12.12% of the total population). The first of these tracts (127.00) lies in the eastern part of Tuscaloosa, a metropolitan area. The second (102.02) is in the smaller adjacent city of Northport. Three other census tracts (two in the eastern part of the county and a third in the western part of the county) showed between 2.95% and 4.67% of Latinos/as/x. This map demonstrates that a significant proportion of the Latino/a/x population in Tuscaloosa County live in the more rural areas surrounding the Tuscaloosa city limits.

Figure 2 of Appendix I, map 2a, shows the Latino/a/x population by census tract alongside family practitioners. These are indicated by a cross if they were identified from the BCBS database and by a star if they were identified from the Medicaid database for all of Tuscaloosa County. Map 2a in Appendix I shows most of the providers clustered in the Tuscaloosa city center. Figure 2 of Appendix I, map 2b, provides an enlarged view of the geographical center of Tuscaloosa County, city of Tuscaloosa. It illustrates the public transit routes alongside the locations of family practitioners and the distribution of the Latino/a/x population. Some of the census tracts with the highest concentrations of Latinos/as/x lacked family care practitioners entirely. In addition, most family practitioners, particularly those located north of the city center, were unreachable by the limited transit routes, which are concentrated in the city of Tuscaloosa.

Maps 2c and 2d of Appendix II demonstrate the geographic distribution of internal medicine physicians. The first map shows the full county with Latino/a/x population distribution and provider locations, and the second shows an enlarged view version of the map within the Tuscaloosa city limits, including the original elements plus public transit routes. In this pair of maps, it is once again clear that internal medicine physicians are clustered in the city center, with a large proportion being unreachable via public transit. The areas with the highest concentrations of Latinos/as/x lack internal medicine providers entirely.

Maps 2e and 2f of Appendix II show how pediatricians are geographically distributed in Tuscaloosa County. Most were in the Tuscaloosa city center, with none operating in the areas with the highest density of Latinos/as/x. Approximately half of the pediatricians were not reachable via public transit.

Finally, the map in Figure 3 of Appendix I illustrates driving times to hospitals in the area. This map includes a wider view of Tuscaloosa County (in the center), along with surrounding counties (Jefferson, Walker, Fayette, Pickens, Greene, Hale, Bibb, and Shelby). Hospitals are indicated by an “H” and varying grayscale shading illustrates the travel times for Latinos/as/x in Tuscaloosa County to drive to those hospitals (from 5 minutes [white] to ≥60 [darkest gray]). The Latino/a/x population in Tuscaloosa County is illustrated by black dots corresponding to different population sizes. The map shows that a significant proportion of the Latino/as/x in Tuscaloosa County must drive 45 or 60 minutes or more to arrive at a hospital.

## Discussion

Our analyses suggest that there are significant gaps in GHA to primary care providers and hospitals for Latinos/as/x living in Tuscaloosa County, Alabama. Specifically, this analysis demonstrated the following:

- The areas where Latinos/as/x live have low proportions of primary care providers.
- There is a dearth of public transit options connecting the areas where Latinos/as/x live to primary care providers.
- Many Latinos/as/x living in Tuscaloosa County have long travel times to hospitals.

These factors point to a challenging landscape with regard to GHA for Latinos/as/x in western Alabama. Since 2010, Alabama has experienced a number of hospital closures in rural areas,^40^ which have contributed to these gaps in GHA to hospital-based care in the region.

It also is important to consider that Latino/a/x in the US South often are members of mixed immigration status households (ie, different members of the same household may have different immigration statuses).^41^ By showing that a significant number of Latinos/as/x in Tuscaloosa County, Alabama, must drive significant distances to reach hospitals and do not have access to alternative viable public transportation, we see that these communities face barriers in attempting to access primary preventive and curative care. Unlike other states, the state of Alabama limits foreigners’ abilities to obtain drivers licenses (including those with legal status such as those with U-visas and Deferred Action for Childhood Arrivals.^42^ As such, traveling long distances to receive care exposes Latinos/as/x and immigrants broadly to negative legal ramifications (ie, traffic citations for driving without a license and possible immigrant detention/deportation).

Outreach and technology-based approaches, including home visit programs,^43^ mobile health units,^44^ and telemedicine,^45^ may be particularly important in bridging the GHA gaps for this and other largely rural populations in the southeastern United States. Various studies have shown that telemedicine has great, largely unfulfilled, potential to facilitate healthcare access among Latinos/as/x in rural areas.^45,46^ Some of this potential was unlocked during the coronavirus disease 2019 pandemic.^47^ It is important that these gains are leveraged toward sustainable healthcare access initiatives for rural Latino/a/x populations. Mobile health clinics also have been shown to be feasible and well-accepted interventions to bridge healthcare access among Latinos/a/x, particularly when paired with community-engaged approaches and local healthcare–community group partnerships.^44,48^ For instance, in their community-engaged study of Latino/a/x farmworkers in California, Tulimiero and colleagues found that offering services in the communities where Latinos/as/x live, providing clinics outside of regular business hours, and encouraging providers to immerse themselves in their patients’ communities to better understand their healthcare needs were effective ways of addressing healthcare challenges among rural Latino/a/x communites.^48^ Importantly, any intervention efforts with this population should account for the significant proportion of Latinos/as/x who are underinsured/uninsured in the United States. Policy-level efforts to ensure access to primary care will be necessary to fully address health disparities among this underserved group.^8,49^

This study had limitations. First, empirical evidence suggests that Latino/as/x are consistently undercounted in census data^50^; therefore, it is possible that we underestimated the population density of this group in Tuscaloosa County, Alabama, as of 2018. Furthermore, we chose 2018 ACS Census dataset as the study base because of its data integrity, whereas the 2019 ACS census dataset and the 2020 decennial census partially lack information regarding the Latino/as/x population in Alabama and Tuscaloosa County. Second, we did not have access to primary care provider or utilization data by Latinos/as/x in Tuscaloosa County. Future research should seek to address these aspects of GHA to better understand the needs of this group. It is worth noting that our research group’s previous qualitative research with Latinos/as/x in western Alabama confirms that the lack of reliable public transportation is a commonly cited barrier to healthcare access for Latinos/as/x in Tuscaloosa County.^8,9^ Finally, although unlikely, it is possible that some providers who do not accept BCBS (BCBS holds 94% of the private insurance industry in Alabama) but do take other private insurers were unaccounted for in these analyses. Despite these limitations, we believe that this work adds to the literature by illustrating GHA challenges for Latinos/as/x in the rural US South. It is important for healthcare providers, researchers, and policy makers working in the US South to be aware of these barriers to care as they work to meet the needs of the newly established Latino/a/x populations in this region.

## Figures and Tables

**Figure 2a.**
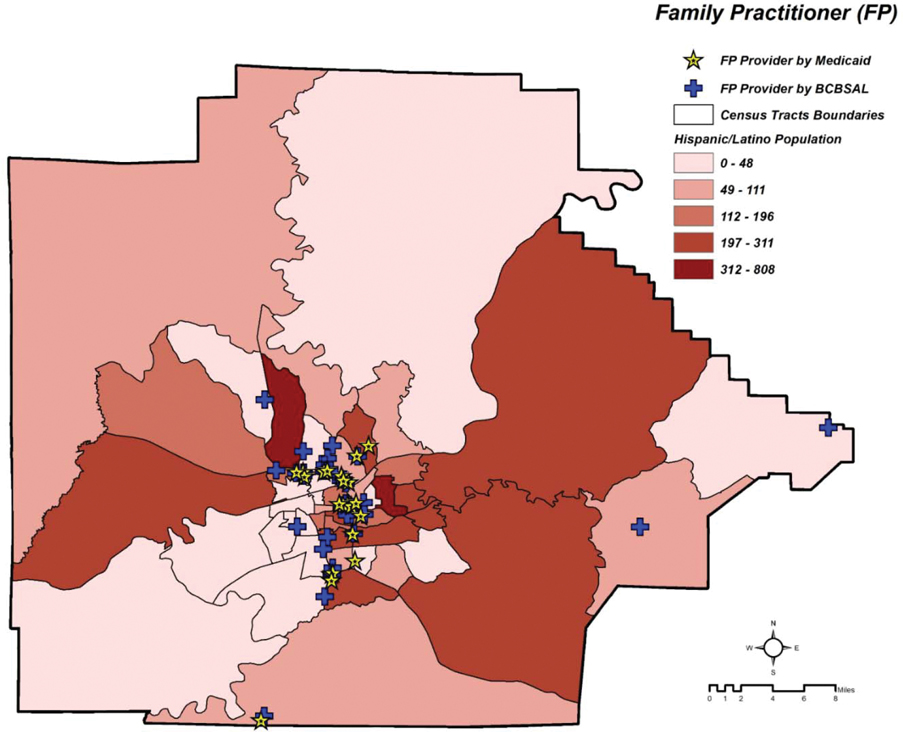
Family Practitioners

**Figure 2b.**
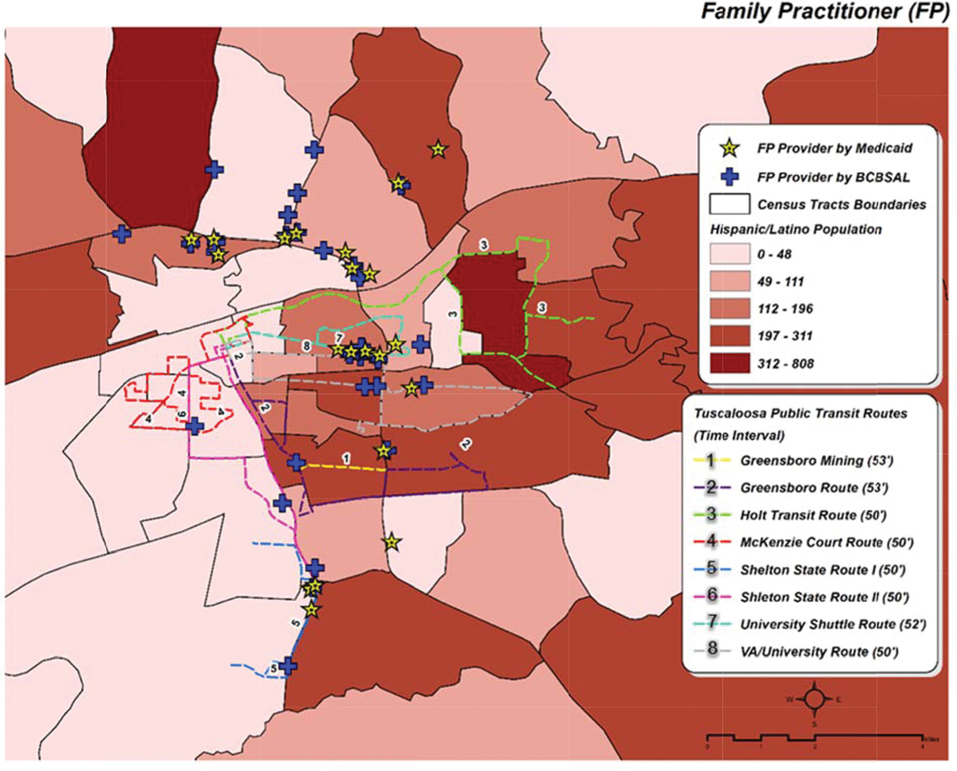
Family Practitione rs and Public Transit Routes

**Figure 2c.**
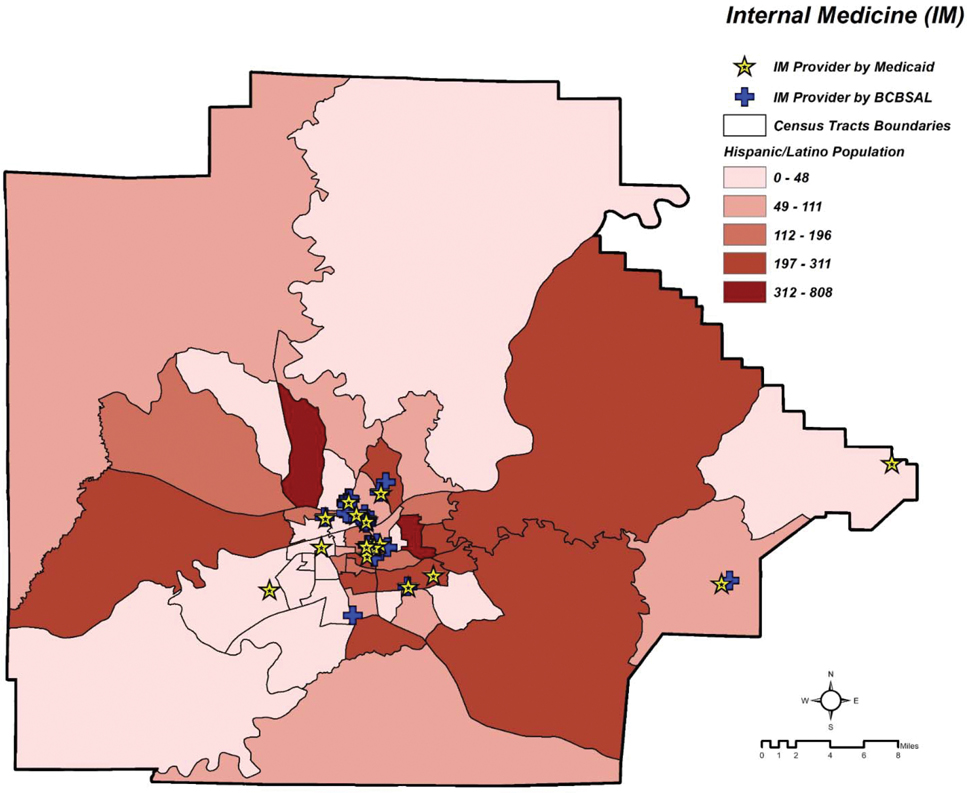
Internal Medicine

**Figure 2d.**
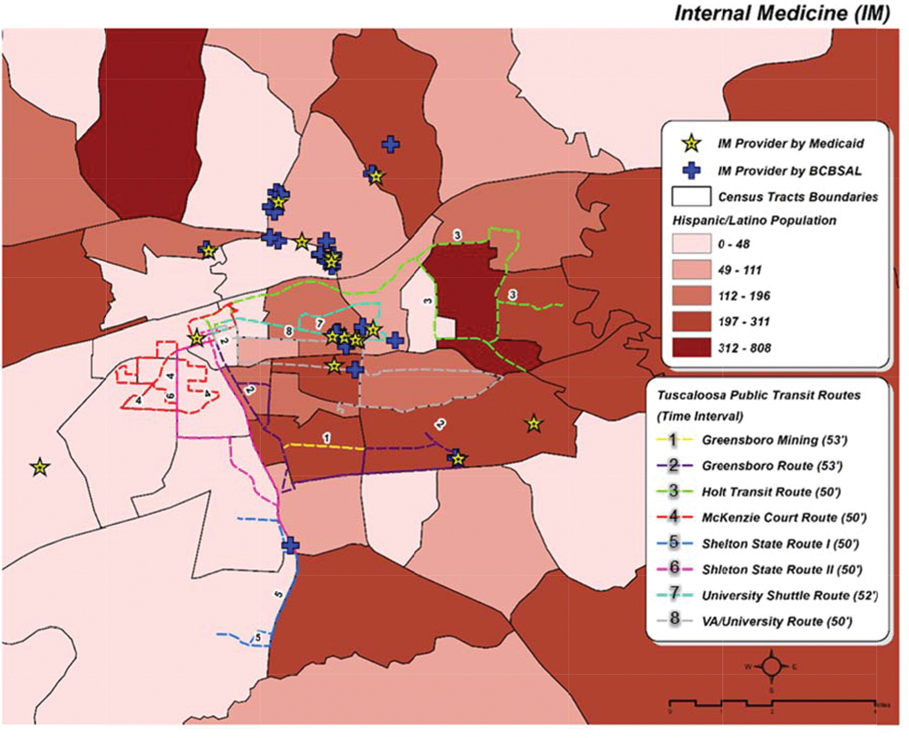
Internal Medicine and Public Transit Routes

**Figure 2e.**
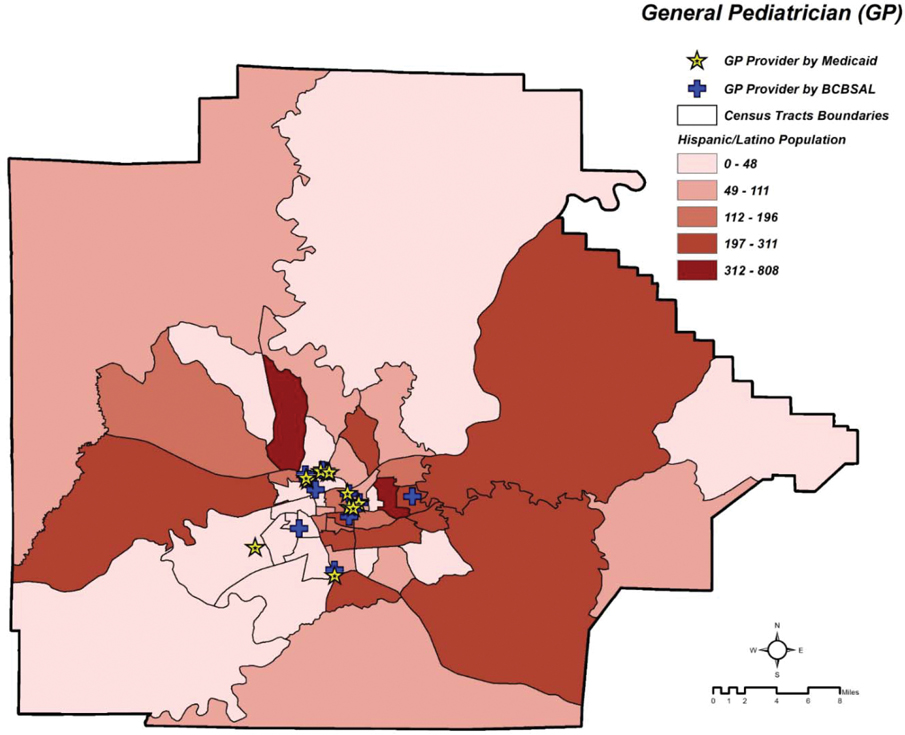
General Pediatricians

**Figure 2f.**
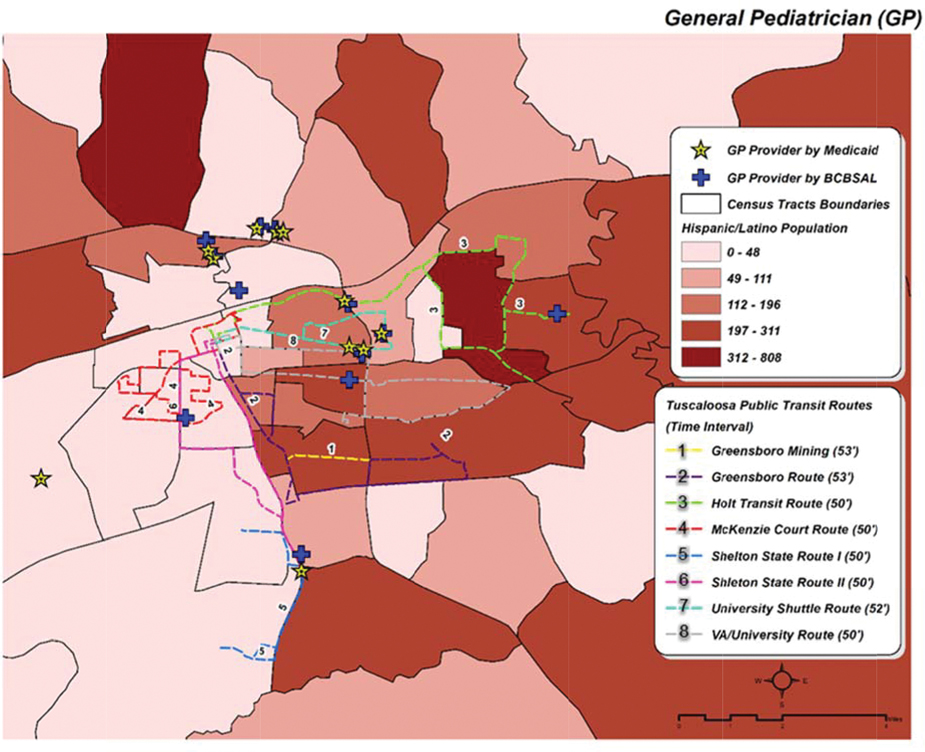
General Pediatricians and Public Transit Routes

## Key Points

- This article describes the availability (defined as number of provider locations) and accessibility (defined as travel impedance between potential patients and provider locations) of primary healthcare and hospital services in our study region in the US South.
- This article identifies geographic healthcare gaps for Latino/a/x populations in this region.
- We discuss potential strategies to address these healthcare access challenges.

## Appendix II.

**Table 1.** Demographic Statistics Summary of Hispanic Population in Alabama, 2014–2018

Demographic Factors	State Level		County Level	

	Counts	Percentage (%)	Counts	Percentage (%)

Population	*203,146*	-	*7,437*	-
Age Group				
0 – 19	*86,378*	*43%*	*2,985*	*40%*
20 – 34	*49,521*	*24%*	*2,232*	*30%*
35 – 54	*48,803*	*24%*	*1,596*	*21%*
55 – 64	*10,310*	*5%*	*351*	*5%*
65 +	*8,134*	*4%*	*273*	*4%*
Below Poverty	*64,845*	*32%* *	*1,827*	*25%* *
Bachelor’s degree or higher	*12,268*	*6%*	*380*	*5%*
Speak English “not well” or “not at all”	*58,938*	*29%*	*2,033*	*27%*
Median household income (in 2018 inflation-adjusted dollars)	*$37,412*	-	*$23,212*	-

Note.

An '-' entry in the estimate column indicates that the statistic is not applicable for this column.

* % of Total Population.

**Table 2.** Health Insurance Coverage of Hispanic Population in Alabama and Tuscaloosa County, Alabama, 2014–2018

Insurance Coverage	Alabama		Tuscaloosa County	

	Counts	Percentage (%)	Counts	Percentage (%)

Population	*203,146*	-	*7,426*	-
Insured	*143,436*	*71%*	*5,370*	*72%*
Uninsured	*59,710*	*29%*	*2,056*	*28%*

Note.

An '-' entry in the estimate column indicates that the statistic is not applicable for this column.

**Table 3.** Providers by Insurance Type by Specialty

Year	Insurance Type	Specialty	# of Providers
2018	Medicaid	General Pediatrician	11
		Internal Medicine	19
		Family Practitioner	25
	BCBS AL	General Pediatrician	13
		Internal Medicine	26
		Family Practitioner	34

Source: Alabama Medicaid Program, BCBS National Doctor and Hospital Database
